# Influence of age and education on the processing of clustering and switching in verbal fluency tasks

**DOI:** 10.1590/1980-57642018dn12-040004

**Published:** 2018

**Authors:** Andressa Hermes Pereira, Ana Bresolin Gonçalves, Maila Holz, Hosana Alves Gonçalves, Renata Kochhann, Yves Joanette, Nicolle Zimmermann, Rochele Paz Fonseca

**Affiliations:** 1 Master student, Universidade Federal do Rio Grande do Sul, RS, Brazil; 2 Psychology undergraduate student, Pontifícia Universidade Católica do Rio Grande do Sul, RS, Brazil; 3 PhD student, Pontifícia Universidade Católica do Rio Grande do Sul, RS, Brazil; 4 PhD, Pontifícia Universidade Católica do Rio Grande do Sul, RS, Brazil; 5 PhD, Centre de Recherche de l’Institut Universitaire de Gériatrie de Montréal, Canada; 6 PhD, Instituto Estadual do Cérebro Paulo Niemeyer, Rio de Janeiro, RJ, Brazil.

**Keywords:** verbal fluency, clustering, switching, age effect, educational level effect, fluência verbal, agrupamento, comutação, efeito de idade, efeito da escolaridade

## Abstract

Verbal fluency (VF) is a widely used tool in neuropsychological assessment.

**Objective:**

We aimed to investigate the influence of age and educational level on clustering and switching in three VF modalities: phonemic (PVF), semantic (SVF) and unconstrained (UVF). We evaluated type of cluster, mean cluster size, and quantity of clusters, intersections, and returns. A total of 260 healthy subjects were assessed.

**Methods:**

Participants were divided into three age groups: young adults (18 to 39 years), middle-aged adults (40 to 59 years) and older adults (60 to 80 years) and into two groups of educational level: 1-8 years (low), 9 years or more (high). A two-way ANOVA analysis was conducted to analyze the effect of age and educational level and its interactions. A repeated measures ANOVA was performed to verify the performance during the task.

**Results:**

A main effect of age was detected on the UVF and SVF scores for total switches, taxonomic clusters, and for the total semantic clusters on the SVF. There was a greater effect of educational level on total switches (UVF, PFV and SVF), taxonomic clusters (UVF and SVF), thematic clusters and total semantic cluster (UVF), phonemic and mixed clusters (PVF), mean cluster size (UVF and SVF) and intersections (SVF). Educational level had a greater effect on all three VF tasks.

## RESUMO

Fluência verbal (FV) são ferramentas amplamente utilizadas na avaliação neuropsicológica.

**Objetivo:**

Nosso objetivo foi investigar a influência da idade e do nível de escolaridade no agrupamento e alternância em três modalidades de fluência verbal: fonêmica (FVF), semântica (FVS) e livre (FVL).

**Métodos:**

Avaliamos o tipo, tamanho médio e quantidade de agrupamentos, alternâncias, intersecções e retornos. Foram divididos 260 indivíduos, em três grupos etários: jovens adultos (18 a 39 anos), adultos de idade intermediária (40 a 59 anos) e idosos (60 a 80 anos) e dois grupos de escolaridade 1-8 (baixa), 9 ou mais (alto). Uma análise ANOVA de dois fatores foi conduzida para analisar o efeito da idade e do nível educacional e suas interações, além de uma ANOVA de medidas repetidas para verificar o desempenho ao longo da tarefa.

**Resultados:**

Encontrou-se efeito principal da idade nas tarefas de FVL e FVS nos seguintes escores: total de alternâncias, agrupamento taxonômicos e no total de agrupamentos semânticos na FVS. Houve um efeito principal do nível educacional no total de alternâncias (FVL, FVF e FVS), agrupamento taxonômicos (FVL e FVS), agrupamento temáticos e cluster semântico total (FVL), clusters fonêmicos e mistos (FVF), tamanho médio de cluster (FVL e FVS) e, finalmente, interseções (FVS). O nível educacional teve efeito maior nas três tarefas de FV.

Verbal fluency (VF) tasks are fast-to-apply low-cost tools widely used in neuropsychological settings. In these tasks, the individual needs to evoke as many words as possible in a given time period (usually one minute) following a given category, such as in the semantic VF (SVF) (fruits, animals, clothes) or phonemic VF (PVF) (letters p, m, f, a, s) tasks. In general, most studies use a quantitative analysis based on the number of correct words generated^1^ and on output evolution over time.^2^

Despite application advantages, most studies still predominantly use general scores which may be uninformative about the nature of the deficit or skill.^3^ Combining this traditional quantitative analysis with a more innovative qualitative analysis, contributes to a richer understanding of the cognitive process intrinsic to VF tasks. Therefore, in order to assess executive functions, lexical-semantic processing and semantic memory cognitive domains, the evaluator must consider the search strategies and the flexibility skills used by the participant to access different lexical and semantic groups.^4^^-^ 7

To this end, a few qualitative models have been created to investigate the cognitive strategies used during VF tasks and the different cognitive processes involved.^3^^,^ 8 ^,^ 9 One of these models proposes that two qualitative components may have a greater influence on the quantitative score.^3^ These are: (a) Clustering, which refers to the generation of words within a given subcategory and involves verbal memory and storage capacity; (b) Switching, which refers to the ability to switch to a new category when a subcategory has been exhausted. Cluster size (number of words that form a cluster) seems to depend on specific cognitive functions, while the cluster and switch initiation capability involves more complex cognitive processes such as cognitive flexibility. Both components have the same relevance for adequate performance on VF tasks.^3^

Several studies have investigated the impact of different neurological clinical features on clustering and switching processing, such as in Parkinson’s disease,^10^^,^ 11 Huntington’s Disease,^12^ multiple sclerosis,^10^ frontal lobe damage,^13^ and temporal lobe epilepsy.^14^ Other studies have centered on psychiatric and neurodevelopment conditions, such as schizophrenia and autism, respectively,^15^^-^ 17 and also on systemic clinical conditions, such as HIV.^18^

There are also studies of healthy children that investigated the relationship of age-related differences in clustering and switching processing.^19^^-^ 21 Regarding healthy adult studies, some studies have sought to investigate the relationship between qualitative variables (such as education, sex and age) and the number of switches and clusters.^22^^-^ 24

Lanting et al.^23^ investigated the effect of age and sex on these two qualitative components; yet they failed to investigate the impact of educational level and not all age groups were included in their study. They found that young adults produced more words overall and switched more often, but had similar clustering production as older subjects. Regarding the effect of sex, males produced larger clusters and females switched more often. Other studies investigating the effect of age found that young adults produced more switches during SVF^23^^,^ 25 ^-^ 27 and PVF^23^^,^ 26 tasks when compared to older adults. There was also a significant difference between groups with different levels of education in the number of switches.^25^^-^ 27 Regarding the average size of clusters, people with a higher educational level had a larger mean cluster size on the PVF task.^26^

In all these studies, only the SVF and PVF modalities with a duration of less than two minutes tasks were used. The influence of individual and sociocultural factors was not explored using the unconstrained VF task (UVF) modality. Finally, none of these studies considered the occurrence of returns to a previously used cluster strategy. Return is a qualitative strategy that requires both episodic and working memory capacity to remember previous category and words evoked and cognitive flexibility to conduct a mental search for others words in the same category.

Given that^1^ clustering and switching, when explored, have involved tasks with a duration of less than two minutes,^2^ that there are no studies with unconstrained modality, and finally^3^ that there are no studies covering all age groups, our primary objective was to verify the role of age and educational level, as well as the interaction between these two variables, regarding the processing of clustering, switching, intersections and returns in three VF modalities (SVF, PVF, UVF).

We hypothesize that adults with higher educational level generate a greater number of switches and returns due to possibly having greater cognitive reserve and executive processing skills. In addition, this group probably forms a larger number of longer intersections and clusters due to the semantic knowledge and identification of the different meanings that the same word can have, thus including them in different clusters. On the other hand, it is believed that older adults with low educational level have greater difficulty in generating new categories and consequently perform fewer switches, due to age-related decline in EF skills.

## METHODS

### Ethical and data collection procedures

The present study was approved as part of a larger project by the Research Ethics Committee of the Pontifical Catholic University of Rio Grande do Sul, number 1679/09. All participants signed a Free and Informed Consent Form. All patients were assessed by trained health professionals with formal experience in neuropsychology. This assessment included an expanded neuropsychological evaluation which occurred in two to three meetings.

The instruments used were part of a broader neuropsychological evaluation and communication battery that was being normalized and adapted for adults. VF tasks were administered in different orders, to control learning effects, strategy creation and sequencing. The VF tasks were transcribed and scored by three independent investigators.

All the evaluators were previously trained to evaluate clustering and switching in VF tasks by a fourth researcher. After training, seven cases of the total sample were evaluated by all judges for final standardization of corrections. The concordance among the three judges was between 96.55 and 100% in blind analyses.

### Study sample

Two hundred and seventy-one healthy subjects were recruited for the present study. The study included subjects aged between 18 and 80 years, without diagnosis of psychiatric or neurological disease, who could read and write and did not have serious hearing problems.

Unfortunately, some participants were excluded for not understanding the tasks (n=2), for interrupting the assessment (n=4), for being older than 80 years of age (n=1), for psychiatric/neurological disease hypothesis (n=1), for having a total score on the Mini-Mental State Examination (MMSE) lower than the cut-off points of Kochhann et al.^28^ (n=2) and, finally, for performing the task in a jocular way (n=1). Thus, a total of 260 participants of both sexes were included.

The participants were divided into three age groups: young adults (18 to 39 years), middle-aged adults (40 to 59 years) and older adults (60 to 80 years). Each of these groups was divided into low (1 to 8 years of formal education) or high (9 years or more of formal education) educational level. Performance on VF tasks did not differ significantly between men and women.

**Table 1:** Sociodemographic characteristics of the sample (n=260).

Variables	Mean (SD)	Minimum-Maximum
Age	43.9 (18.4)	18-80
Educational level	11.7 (6.1)	02-35
MMSE	28.2 (1.9)	22-30
Economic Class	27.2 (10.3)	06-58
	**% (n)**	
Sex (female)	71.5 (186)	

MMSE: Mini-Mental State Examination.

### Instrument

VF subtests from the *Protocole Montréal d’Évaluation de la Communication – Protocole MEC* 29 adapted to Brazilian Portuguese^30^ were employed, where the participant must evoke the highest number of words (within a given category) over a certain period of time. In the PVF task, the participants must evoke as many words as possible beginning with the letter P within 2 minutes. For the SVF task, participants have to produce as many items of clothing within two minutes. Finally, on the UVF task, the participant must evoke as many words as possible without any category constraints in two and a half minutes. We analyzed the total number of correct answers on the entire task, and for intervals of 30 seconds, as well as clustering, switching, cluster types, intersections and returns variables.

### Clustering and switching analysis

Clustering and switching analysis was conducted according to the methods proposed by Troyer,^27^ Abwender et al.,^31^ and according to Gonçalves et al.’s manual.^32^ Finally, we summarized all the information and presented the normative data for adults found in Hermes-Pereira et al.’s manual.^33^ The three VF tasks were scored by mean cluster size, and the number of clusters, switches, returns and intersections produced.

- Cluster: defined as a grouping of two or more words that belonged to the same phonological, semantic (taxonomic, or thematic) or mixed (phonological + semantic) category.
- Phonemic Cluster: considered groupings of words successively evoked that begin with the same first two letters, or the same sound, or that rhyme, or that are homonyms.
- Total semantic clusters: defined as successively evoked words that belong to the same semantic category (taxonomic), or by union of words not restricted to a category, but to an event or specific situation (thematic).
- Number of clusters: characterized by the frequency of clusters on each VF task. Cluster size was calculated from the number of evoked words of the same category minus one. The perseverations and intrusions were included in the cluster size calculation, because they provide the form of strategies used throughout the task.
- Number of switches: number of transitions that the participant made between one cluster and another. Calculated number of clusters – 1.
- Number of returns: characterized by the number of times that the individual returned to a strategy employed previously.
- Intersections: the ability to use words from a cluster as a “trigger” to start the next (example: cluster 1: hand, arm, head, cluster 2: notebook, camel, shirt, in this case the last word of cluster 1 classified as semantic taxonomy served as a connection for the next cluster, classified as phonological). A Brazilian Portuguese example follows for more clarity, cluster 1: blusa, casaco, jaqueta, bota; cluster 2: bola, futebol, campo, jogadores.

### Data analysis

A Kolmogorov-Smirnov test was performed to assess the normality distribution of the data. Although the distribution was non-parametric, we used parametric analyses in order to verify the interactions. This analyses was elected because the majority of the variables had a skewness value below 2, with the exception of mean cluster size (UVF all blocks and SVF block 3), total cluster size, total phonemic clusters and total mixed clusters (UVF and SVF), and total intersections (SVF). Gravetter and Wallnau (2014) suggest that the skewness must range between (±2) to be considered a normal distribution.

Thus, a two-way ANOVA was conducted to analyze the effect of age and educational level, as well as the possible interactions between these two factors, in all three VF modalities (SVF, PVF, and UVF) on the following scores: switches, mean cluster size, types of clusters, intersections, and returns; followed by Bonferroni’s post-hoc analysis. Repeated measures ANOVA of the switches were performed to verify the performance over the time intervals in all VF modalities. For all analyses, differences with significance level p≤0.01 were considered significant.

## RESULTS

Tables 2 and 3 show the ANOVA results for the main effects of age and educational level, respectively. Values of descriptive analyses by different age groups and educational levels are found in a chapter by Hermes-Pereira et al.^33^

**Table 2:** ANOVA age effect.

Variables	F	p	n^2^
Total switches UVF	6.845	0.001	0.056
Total mean cluster size UVF	2.846	0.06	0.024
Taxonomic cluster UVF	6.207	0.002	0.051
Thematic cluster UVF	0.999	0.37	0.009
Total semantic cluster UVF	5.22	0.006	0.043
Phonological cluster UVF	3.086	0.048	0.026
Mixed cluster UVF	1.173	0.311	0.01
Intersections UVF	0.773	0.463	0.007
Returns UVF	0.071	0.931	0.001
Total switches PVF	2.489	0.085	0.02
Total mean cluster size PVF	2.528	0.082	0.02
Taxonomic cluster PVF	0.338	0.713	0.003
Thematic cluster PVF	0.996	0.371	0.008
Total semantic cluster PVF	0.406	0.667	0.003
Phonological cluster PVF	0.96	0.384	0.008
Mixed cluster PVF	2.759	0.065	0.022
Intersections PVF	2.239	0.109	0.018
Returns PVF	0.617	0.541	0.005
Total switches SVF	9.467	0.001	0.071
Total mean cluster size SVF	0.28	0.756	0.002
Taxonomic cluster SVF	8.996	0.001	0.068
Phonological cluster SVF	2.366	0.096	0.019
Mixed cluster SVF	0.252	0.777	0.002
Intersections SVF	1.435	0.24	0.011
Returns SVF	3.136	0.045	0.025

Degrees of freedom UVF 2.231, PVF 2.250 and SVF 2,247. UVF: unconstrained verbal fluency; PVF: phonemic verbal fluency; SVF: semantic verbal fluency.

**Table 3:** ANOVA educational level effect.

Variables	F	p	n^2^
Total switches UVF	35.587	0.001	0.137
Total mean cluster size UVF	5.925	0.016	0.137
Taxonomic cluster UVF	16.56	0.001	0.067
Thematic cluster UVF	31.027	0.001	0.118
Total semantic cluster UVF	34.443	0.001	1.13
Phonological cluster UVF	1.736	0.189	0.007
Mixed cluster UVF	0.184	0.669	0.001
Intersections UVF	6.467	0.012	0.027
Returns UVF	2.086	0.15	0.009
Total switches PVF	29.726	0.001	0.106
Total mean cluster size PVF	3.009	0.084	0.12
Taxonomic cluster PVF	1.422	0.234	0.006
Thematic cluster PVF	6.142	0.014	0.024
Total semantic cluster PVF	4.707	0.031	0.018
Phonological cluster PVF	17.463	0.001	0.065
Mixed cluster PVF	15.494	0.001	0.058
Intersections PVF	0.068	0.794	0
Returns PVF	4.85	0.029	0.019
Total switches SVF	47.3	0.001	0.161
Total mean cluster size SVF	12.663	0.001	0.049
Taxonomic cluster SVF	55.599	0.001	0.184
Phonological cluster SVF	1.197	0.275	0.005
Mixed cluster SVF	4.655	0.032	0.018
Intersections SVF	6.393	0.012	0.025
Returns SVF	1.316	0.252	0.005

Degrees of freedom UVF 1.231, PVF 1.250 and SVF 1.247. UVF: unconstrained Verbal Fluency; PVF: phonemic verbal fluency; SVF: semantic verbal fluency

### Age effect

Table 2 shows that the group of young adults performed better than the group of older adults on the total number of taxonomic clusters and total semantic clusters (p=0.002, p=0.004, respectively) on the UVF tasks. On the SVF, the group of older adults had worse performance than the other two groups on the total number of taxonomic clusters (p<0.001 for young adults, p=0.010 for middle-aged adults).

In relation to the total number of words, on both UVF and SVF, the young adults (p<0.001, UVF, p<0.001, SVF) and middle-aged groups had better performance than the older adults group (p=0.001, UVF; p<0.001, SVF). Regarding the number of switches, the older adults had worse performance than the young adults (p=0.001) on the UVF, and the other two groups on the SVF (p<0.001 for young adults; p=0.006 for middle-aged adults).

### Educational level effect

For total switching, on all fluency modalities, the low educational level group had worse performance than the high educational level group. For total mean cluster size, the high educational level group had a smaller cluster size than the low educational level group on the UVF and SVF.

Regarding cluster types on the UVF, for thematic clusters, taxonomic clusters and total semantic clusters, the group with higher educational level performed better than the group with low educational level. A similar pattern of performance also occurred on the PVF. For the variables thematic clusters, phonemic clusters and mixed clusters, the group with higher educational level performed better than the group with low educational level. Finally, on the SVF, for total taxonomic clusters, the group with higher educational level also performed better than the group with low educational level.

### Interactions between age and educational level

Interactions between age and educational level occurred only on the following scores: (1) total mean cluster size on UVF; (2) total intersections on the SVF. Middle-aged adults with low educational level had worse performance in relation to middle-aged adults with high level educational level while young adults with high educational level had better performance on VF tasks.

### Repeated measures between intervals

UVF – Interval * age (F (2.20,43)=4.53, p=0.012, n²=0.03) with young adults showing higher switching productivity on the four initial blocks compared to the older adults (p<0.001). There was no interaction between interval * educational level.

PVF – on this task, there was no interaction between interval * age nor between interval * educational level. The lower productivity of switches during the last 30 seconds of the task compared to the others occurred independently of age and educational level of the participants (F (1.32,05)=28.46, p<0.001, n^2^=0.10).

SVF – There was interaction between age * interval (F (2.8,22)=8.08, p<0.001, n²=0.06) with older adults producing significantly fewer switches than young adults and middle-aged adults only on block 1 (post hoc p<0.001, p=0.001, respectively). Similarly, interaction between educational level intervals (F (1.16.22)=16.02, p<0.001, n²=0.06) was observed, where adults with high educational level were more productive on all blocks (p<0.002), with the exception of block 4.

## DISCUSSION

The present study sought to investigate the effects of age and formal educational level on three VF task modalities in a sample of healthy individuals. Clusters, switches, type of clusters, intersections and returns were investigated for all three modalities. Educational level influenced a greater number of variables than age. Interaction between both variables occurred only in the mean cluster size on the unconstrained VF and in total intersections for semantic VF, with effect of educational level on the middle-aged group. On the UVF, young adults outperformed the older adults group in number of switches on the four initial blocks, but not on the last block. On the PVF, all age groups had fewer switches on the last block. Finally, on the SVF, there were age differences between young and middle-aged groups in switching on block 1, and educational differences in productivity for all blocks, except the last block.

### Effect of age

The results of the present study indicate that age is a factor that impacts performance for the following variables: total switches, total taxonomic clusters and total semantic clusters (taxonomic and thematic) on the UVF, and the total switches and total taxonomic clusters on the SVF. The ability to switch can be considered an executive processing strategy, since the individual needs to organize and change their strategy throughout the task. Therefore, it seems easier to observe the effect of age-related executive decline in tasks involving semantic abilities such as the UVF and SVF. Age effect was not found in the PVF modality, which is a task less dependent of semantic memory when compared to SVF and UVF.

On the SVF, age had a predictive effect on total taxonomic clusters. This may be explained by the age-related difficulty accessing semantic information, especially in a stressful context, such as pressure to enunciate words within a limited time, and therefore older adults would need to resort to a larger number of switches. Akin to Troyer et al., we found no association between age and mean cluster size on the PVF. 27 This modality task seems to require greater executive functioning skills, and therefore is a more difficult task for all age groups.

### Effect of educational level

Educational level played an important role in the performance of groups on all three VF tasks, highlighting the importance of investigating and considering sociocultural aspects in neuropsychological assessments.^34^ Educational level influenced ten variables of the total 25 investigated. On the UVF, variables influenced were total switches, total thematic and taxonomic clusters, and total semantic clusters (thematic and taxonomic). On the PVF, total number of switches, in addition to phonemic and mixed clusters, were influenced by educational level. While on the SVF, educational level influenced total switches, mean cluster size and taxonomic cluster.

Education seems to play an important role in the strategies used during all VF modality tasks. Clinicians should be especially attentive to individuals with low educational level during neuropsychological assessment to avoid cases of false positive deficits. This may occur because clinicians could infer that the patient is presenting executive dysfunction, mnemonic difficulty or linguistic impairments, when in fact the results are actually reflecting a lack of cognitive stimulation during their educational process.^35^

Troyer et al.^3^ suggested that mean cluster size correlates with increase in vocabulary size over the course of life. In our findings, education influenced mean size on the SVF. The semantic modality task requires both EF and semantic memory, and therefore may be influenced by the stimuli involved during the educational processes and semantic knowledge acquisition.

Thematic and the taxonomic cluster type on the UVF were influenced by educational level. The first cluster type is associated with learning, and the words were linked through mental images associated with each other over time or space,^36^ while the latter requires more information planning capacity from semantic memory.^25^ Neither educational level nor age had an impact on phonemic cluster type.

In addition, on the UVF, educational level also tended to influence the number of intersections. The high educational level group had a greater number of intersections than the low-educated group. Intersection refers to the capacity to simultaneously form two clusters. This capacity is associated with the episodic buffer component of working memory. This association helps the participant retain the previously integrated information and, at the same time, to be flexible to search for a new cluster, Hence, this effective strategy may be associated with cognitive reserve (CR) capacity, educational stimulation, and cognitive flexibility.

A recent review defines cognitive reserve as the adaptability of cognitive processes that help us understand the differential susceptibility of cognitive abilities or day-to-day functioning to brain aging, pathology, or insult.^37^ Differences in CR are influenced by the interaction of innate individual differences and lifetime exposures across lifespan. A highly relevant lifetime exposure factor seems to be formal education.^37^

### Interaction effects between age and educational level

There was interaction between age and educational level for only two variables (mean cluster size on the UVF and total intersections on the SVF). In both cases, middle-aged adults with low educational level had worse performance in relation to middle-aged adults with high educational level. The high educational group had more effective performance on VF tasks. Thus, in this age group, we can observe the key role of educational stimulation in mnemonic and linguistic processing. More specifically, there is a greater ability to explore the same topic, as well as a more effective chaining process among the topics for task performance.

### Performance during tasks

There was no interaction between age and educational level during the tasks. Our analysis also shows that differences found in switching and productivity during the tasks were not evident on the last blocks. Thus, greater difficulty switching and evoking words on the final task seems to be an overall difficulty on VF tasks.

In conclusion, our data highlights the importance of qualitative analyses for the clinical application of these three VF modality tasks. They are widely used tasks and considered sensitive for executive capacity, but whose potential remains underexplored. We suggest that future studies investigate beyond octogenarians, covering nonagenarians and centenarians. Different clinical, neurological and psychiatric conditions should also be investigated so that neuropsychological diagnoses can include both quantitative and qualitative aspects, enabling a better diagnosis, prognosis, stimulation and neuropsychological rehabilitation. Limitations of the present study were the non-inclusion of important variables, such as independent IQ, vocabulary, EF, and non-verbal assessment, as well as the stratification of educational level into only two groups.

In conclusion, educational level had a greater influence on several VF quantitative variables investigated.^38^ These findings are important for a better understanding of cognitive functioning with different educational levels for Brazilian Portuguese speakers. The fact that age had less influence than educational level highlights the importance of educational level in linguistic, mnemonic and executive processing capacity development.

## References

[1] Alegret M, Peretó M, Pérez A, Valero S, Espinosa A, Ortega G (2018). The Role of Verb Fluency in the Detection of Early Cognitive Impairment in Alzheimer's Disease. J Alzheimer's Dis.

[2] Zimmermann N, Parente MA de MP, Joanette Y, Fonseca RP (2014). Unconstrained, phonemic and semantic verbal fluency: age and education effects, norms and discrepancies. Psicol Reflexão e Crítica.

[3] Troyer AK, Moscovitch M, Winocur G (1997). Clustering and switching as two components of verbal fluency: Evidence from younger and older healthy adults. Neuropsychology.

[4] Davidson PSR, Gao FQ, Mason WP, Winocur G, Anderson ND (2008). Verbal fluency, Trail Making, and Wisconsin Card Sorting Test performance following right frontal lobe tumor resection. J Clin Exp Neuropsychol.

[5] Jungwirth S, Zehetmayer S, Bauer P, Weissgram S, Tragl KH, Fischer P (2009). Prediction of Alzheimer dementia with short neuropsychological instruments. J Neural Transm.

[6] Lezak M, Howieson DB, Bigler ED, Tranel D (2004). Neuropsychological Assessment.

[7] Lezak MD (1995). Neuropsychological assessment.

[8] Smith PT, Claxton GL (1972). Lexical search and phonemic organisation in memory.

[9] Crowe SF (1996). The Performance of Schizophrenic and Depressed Subjects on Tests of Fluency: Support for a Compromise in Dorsolateral Prefrontal Functioning. Aust Psychol.

[10] Tröster AI, Fields JA, Testa JA, Paul RH, Blanco CR, Hames KA (1998). Cortical and subcortical influences on clustering and switching in the performance of verbal fluency tasks. Neuropsychologia.

[11] Troyer AK, Moscovitch M, Winocur G, Leach L, Freedman M (1998). Clustering and switching on verbal fluency tests in Alzheimer's and Parkinson's disease. J Int Neuropsychol Soc.

[12] Rich JB, Troyer AK, Bylsma FW, Brandt J (1999). Longitudinal analysis of phonemic clustering and switching during word-list generation in Huntington's disease. Neuropsychology.

[13] Troyer AK, Moscovitch M, Winocur G, Alexander MP, Stuss DON (1998). Clustering and switching on verbal fluency the effects of focal frontal - and temporal - lobe lesions. Neuropsychologia.

[14] Zakzanis KK, McDonald K, Troyer AK (2011). Component analysis of verbal fluency in patients with mild traumatic brain injury. J Clin Exp Neuropsychol.

[15] Bozikas V, Kosmidis M, Karavatos A (2005). Disproportionate impairment in semantic verbal fluency in schizophrenia: differential deficit in clustering. Schizophr Res.

[16] Moore DJ, Savla GN, Woods SP, Jeste D V, Palmer BW (2006). Verbal fluency impairments among middle-aged and older outpatients with schizophrenia are characterized by deficient switching. Schizophr Res.

[17] Tager-Flusberg H, Paul R, Lord C, Cohen DJ, Volkmar FR (2005). Language and communication in autism. Handbook of Autism and Pervasive Developmental Disorders.

[18] Woods SP, Conover E, Rippeth JD, Carey CL, Gonzalez R, Marcotte TD (2004). Qualitative aspects of verbal fluency in HIV-associated dementia A deficit in rule-guided lexical-semantic search processes?. Neuropsychologia.

[19] Hurks PP, Hendriksen JG, Vles JS, Kalff A, Feron FJ, Kroes M (2004). Verbal fluency over time as a measure of automatic and controlled processing in children with ADHD. Brain Cogn.

[20] Koren R, Kofman O, Berger A (2005). Analysis of word clustering in verbal fluency of school-aged children. Arch Clin Neuropsychol.

[21] Sauzéon H, Lestage P, Raboutet C, N'Kaoua B, Claverie B (2004). Verbal fluency output in children aged 7-16 as a function of the production criterion Qualitative analysis of clustering, switching processes, and semantic network exploitation. Brain Lang.

[22] Kavé G, Kigel S, Kochva R (2008). Switching and clustering in verbal fluency tasks throughout childhood. J Clin Exp Neuropsychol.

[23] Lanting S, Haugrad N, Crossley M (2009). The effect of age and sex on clustering and switching during speeded verbal fluency tasks. J Int Neuropsychol Soc.

[24] Weiss EM, Ragland JD, Brensinger CM, Bilker WB, Deisenhammer E a, Delazer M (2006). Sex differences in clustering and switching in verbal fluency tasks. J Int Neuropsychol Soc.

[25] Brucki SMD, Rocha MSG (2004). Category fluency test: effects of age, gender and education on total scores, clustering and switching in Brazilian Portuguese-speaking subjects. Brazilian J Med Biol Res.

[26] Kosmidis Mh, Vlahou Ch, Panagiotaki P, Kiosseoglou G (2004). The verbal fluency task in the Greek population: Normative data, and clustering and switching strategies. J Int Neuropsychol Soc.

[27] Troyer AK (2000). Normative Data for Clustering and Switching on Verbal Fluency Tasks. J Clin Exp Neuropsychol.

[28] Kochhann R, Varela JS, Lisboa CS de M, Chaves MLF (2010). The Mini Mental State Examination: Review of cutoff points adjusted for schooling in a large Southern Brazilian sample. Dement Neuropsychol.

[29] Joanette Y, Ska B, Côté H (2004). Protocole Montreál d'Évaluation de la Communication.

[30] Fonseca RP, Parente MA de MP, Cote H, Ska B, Joanette Y (2008). Bateria MAC - Bateria Montreal de Avaliação da Comunicação.

[31] Abwender DA, Swan JG, Bowerman JT, Connolly SW (2001). Qualitative Analysis of Verbal Fluency Output: Review and Comparison of Several Scoring Methods. Assessment.

[32] Gonçalves HA, Fonseca FP, Cargnin C, Fonseca RP, Fonseca RP, Liberatore PM, Zimmermann N (2016). Análise quantitativa e qualitativa das estratégias de evocação de palavras em tarefas de fluência verbal para crianças. Avaliação de linguagem e funções executivas em crianças.

[33] Gravetter Frederick J, WallnauLarry B (2014). Essentials of statistics for the behavioral sciences.

[34] Hermes-Pereira A, Gonçalves APB, Holz MR, Gonçalves H, Kochhann R, Joanette Y, Zimmermann N, Fonseca RP (2017). Análises de clustering e switching em tarefas de fluência verbal livre, fonêmica e semântica: dados normativos em adultos. Avaliação de funções executivas e linguagem em adultos.

[35] Passos VM de A, Giatti L, Bensenor I, Tiemeier H, Ikram MA, de Figueiredo RC (2015). Education plays a greater role than age in cognitive test performance among participants of the Brazilian Longitudinal Study of Adult Health (ELSA-Brasil). BMC Neurol.

[36] Mungas D, Reed BR, Farias ST, Decarli C (2010). NIH Public. Access.

[37] Gutchess AH, Hedden T, Ketay S, Aron A, Gabrieli JDE (2010). Neural differences in the processing of semantic relationships across cultures. Soc Cogn Affect Neurosci.

[38] Stern Y, Arenaza-Urquijo EM, Bartrés-Faz D, Belleville S, Cantilon M, Chetelat G (2018). Whitepaper: Defining and investigating cognitive reserve, brain reserve, and brain maintenance. Alzheimer's Dement.

